# Sit still and pay attention: Using the Wii Balance-Board to detect lapses in concentration in children during psychophysical testing

**DOI:** 10.3758/s13428-018-1045-4

**Published:** 2018-05-16

**Authors:** Pete R. Jones

**Affiliations:** 1 Institute of Ophthalmology, University College London (UCL), 11-43 Bath Street, London, EC1V 9EL UK; 2 NIHR Moorfields Biomedical Research Centre, London, UK; 3 City University of London, London, UK

**Keywords:** Psychophysics, Children, Lapse rates, Task engagement, Concentration, Attentiveness, Test development, Wii Fit Balance Board, Postural instability, Center of pressure, Catch trials, Receiver operating characteristic

## Abstract

During psychophysical testing, a loss of concentration can cause observers to answer incorrectly, even when the stimulus is clearly perceptible. Such lapses limit the accuracy and speed of many psychophysical measurements. This study evaluates an automated technique for detecting lapses based on body movement (postural instability). Thirty-five children (8–11 years of age) and 34 adults performed a typical psychophysical task (orientation discrimination) while seated on a Wii Fit Balance Board: a gaming device that measures center of pressure (CoP). Incorrect responses on suprathreshold catch trials provided the “reference standard” measure of when lapses in concentration occurred. Children exhibited significantly greater variability in CoP on lapse trials, indicating that postural instability provides a feasible, real-time index of concentration. Limitations and potential applications of this method are discussed.

**Electronic supplementary material:**

The online version of this article (10.3758/s13428-018-1045-4) contains supplementary material, which is available to authorized users.

Both basic science and clinical practice are often concerned with measuring the limits of perception. For example, the faintest sound a child can hear can be used as a marker for hearing loss, whereas the dimmest light a child can see can be used to study the structure or efficiency of the retina. The psychophysical procedures used to make such measurements generally assume that the observer understands the task instructions, and is attempting to comply with them on every trial. However, many individuals—and children in particular—may struggle to sustain their attention throughout a prolonged period of testing. Their mind may wander, and such lapses in concentration can result in perceptual estimates becoming noisy or biased (see below). The goal of the present work was to assess whether Postural Instability (or “fidgeting”) could be used as an autonomous and inexpensive marker for lapses in concentration in children. This paper also considers how such measurements could be used to improve the accuracy and reliability of psychophysical estimates.

## The problem of lapses in psychophysics

To measure the limits of perception, we typically use psychophysical procedures such as transformed staircases (Levitt, 1971), weighted staircase (Kaernbach, 1991), QUEST (Watson & Pelli, 1983), ZEST (King-Smith, Grigsby, Vingrys, Benes, & Supowit, 1994), PEST (Taylor & Creelman, 1967), Psi (Kontsevich & Tyler, 1999), QUEST+ (Watson, 2017), or similar methods (for reviews, see Kingdom & Prins, 2010; Leek, 2001; Treutwein, 1995). These algorithms operate by presenting stimuli of varying magnitude, and attempting to find the smallest stimulus magnitude to which the observer responds accurately (e.g., detects, discriminates, or identifies). This is the observer’s “threshold,” “limen,” or “just noticeable difference,” and is generally thought of as a pure measure of perceptual ability.

However, to make this inference we must assume, implicitly, that the observer’s responses are determined solely by their perception of the stimulus. In practice, this assumption is seldom correct. Thus, psychophysical procedures are often lengthy and repetitive, and an observer’s mind may sometimes wander. Such a lapse in concentration can cause the observer to forget, momentarily, what the required judgment is or how to respond appropriately. Alternatively, they may fail to fixate the stimulus altogether, and so be forced to guess. In either case, the observer’s response will be independent of the stimulus magnitude, and the probability of answering correctly on that trial will be chance (e.g., 50%, when the task is a two-alternative forced choice; 2AFC).

Lapses in concentration are problematic because they add noise and/or bias to our psychophysical measurements. In the simplest terms, the increase the likelihood of an imperceptible stimulus being reported as seen (false positives), or of a perceptible stimulus being reported as unseen (false negatives). This at best introduces random measurement error. Furthermore, in many experimental designs, the likelihood of responding correctly by chance is lower than the likelihood of responding incorrectly by chance. This means that lapses in concentration can cause the underlying psychophysical algorithm to systematically *underestimate* the observer’s true sensitivity. This tendency is further exacerbated when using traditional “staircase” procedures, which often start at a high (suprathreshold) stimulus magnitude, and require the observer to make a protracted series of correct responses to reach threshold.

**Fig. 1:**
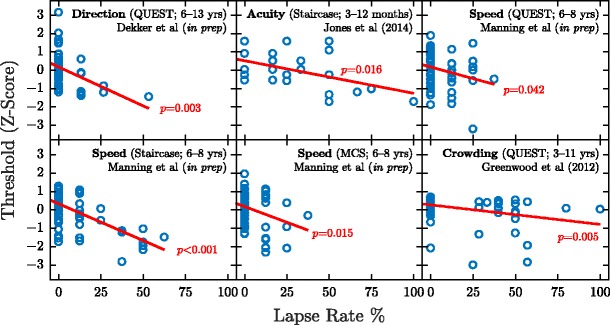
Empirical data from multiple authors/studies (Greenwood et al., 2012; Dekker & McClean, unpublished; Jones, Kalwarowsky, Atkinson, Braddick, & Nardini, 2014; Manning & Pellicano, unpublished), showing the relationship between lapses in concentration and estimated sensitivity. To allow for easy comparisons across studies, the detection/discrimination thresholds within each individual study were converted (independently) to *Z* scores and normalized such that higher values indicated greater sensitivity. Lapses in concentration were measured by lapse rates (false negative responses on suprathreshold catch trials). The circles indicates individual children, and red lines represent orthogonal least-squares fits to the data points. In all six cases, a significant negative association is apparent between lapse rates and estimated sensitivity. Of particular note are the strength of the effect and the fact that it persists robustly across different tasks, psychophysical algorithms, participants, and research groups

Some of the more blatant confounding effects of lapses can be militated against by using using simple heuristics (e.g., “restart the test if an incorrect answer occurs in the first three trials”) or by using advanced psychophysical algorithms that incorporate “nuisance parameters,” explicitly designed to model the likelihood of a “false negative” response occurring (Prins, 2013; Watson, 2017). Unfortunately, neither of these approaches is capable of fully solving the problem in practice, and, as the empirical data in Fig. 1 show, all current methods are liable to be biased nontrivially by substantial numbers of random guesses (see also Manning, Jones, Dekker, & Pellicano, unpublished; Wightman & Allen, 1992; Witton, Talcott, & Henning, 2017)

Lapses in concentration are particularly concerning for investigators working with children, in whom lapses are particularly prevalent (Godwin et al., 2016; Kaunhoven & Dorjee, 2017; Moore, Ferguson, Halliday, & Riley, 2008; Smallwood, Fishman, & Schooler, 2007; Wightman & Allen, 1992; Witton et al., 2017). It is not always clear to what extent this represents a fundamental difference in cognition (e.g., attention short-term memory) and/or reflects the fact that children are often given less practice and are often sampled from a more heterogeneous population than adult psychophysical cohorts. Regardless of the reason, the deleterious effects are clear. For scientists, lapses slow down testing, introduce unnecessary measurement error, and/or cause sensitivity to be underestimated systematically– potentially leading to spurious “developmental differences” between adults and children. For clinicians, high lapse rates can result in “false alarm” referrals that incur direct costs, cause unnecessary worry for parents, and, if they happen regularly enough, can make entire screening programs untenable. These concerns are a key reason why many developmental research question still lack satisfactory answers and why pediatric evaluation remains “as much of an art as a science” (Wilson, 2008).

## Common responses to the problem of lapses

The challenge of maintaining concentration during a psychophysical assessment is widely acknowledged, and is referred to by many names. Thus, investigators often talk of the need for vigilance or sustained attention, or refer to failures in terms of attentional lapses, fussiness, noncompliance, or inattentiveness.

Over the years, investigators have responded to this challenge in different ways. One approach is to shorten test durations by reducing the number of trials. However, sacrificing data in this way can ironically serve to amplify overall measurement error (see Witton et al., 2017). A second approach is to try to make the task as engaging as possible. However, the core content is often dictated by the need for well-defined stimuli, and the procedure is often constrained by the fact that most psychophysical algorithms inherently require a sequence of repetitive judgments. Our ability to make psychophysics intrinsically appealing is therefore limited, and excessive “gamification” can even risk making the experiment unnecessarily long or introduce confounding distractors. A third approach is to rely on advanced psychophysical algorithms to explicitly model and adjust for lapses (see Prins, 2013; Watson, 2017). Given infinite trials and an ideal observer, this approach is highly attractive. However, with limited trials, and observers whose concentration levels may vary markedly between individuals and/or over time, the benefits of these techniques are limited. This is shown empirically in Fig. 1 and has been previously discussed in theoretical terms by Wichmann and Hill (2001). A fourth approach is to simply discard suspicious data and exclude or “replace” (Wichmann & Hill, 2001) observers who exhibit excessive lapses in concentration. However, this is not a viable option in clinical practice and is undesirable in research, as it can lead to poor practices and the obscuring of important individual differences. Finally, then, the response of some investigators has been to counsel despair and advocate abandoning psychophysics in children altogether—for example, in favor of purely neurophysiological measurements (Witton et al., 2017).

Notably, however, some psychophysical tests exist that *are* capable of operating robustly, even in extremely challenging populations. For example, in the visual domain preferential-looking procedures exist for measuring acuity (McDonald et al., 1985; Teller, McDonald, Preston, Sebris, & Dobson, 1986) and visual fields (Fulton, Manning, & Dobson, 1978) in infants, while in the auditory domain head-turn audiometry provides analogous measures of detection thresholds in infants (Day et al., 2002). How is it that these methods are able to function effectively, given that, for example, even a healthy infant will often not respond to a suprathreshold stimulus on around one in three trials (Jones, Kalwarowsky, Braddick, Atkinson, & Nardini, 2015)? The answer, and the distinguishing hallmark of these tests, is that they require an experienced operator to be present throughout testing. The operator is trained to discern whether the child is alert, engaged, and attentive, and is empowered to take appropriate action if a lapse in concentration occurs—typically in the form of rerunning trials, offering encouragement, or initiating short breaks. Perhaps most crucially, the human operator is able to ignore “bad” trials, and in so doing filters them out so that they do not contaminate the underlying psychophysical algorithm.

Unfortunately, as a general solution it is not practical to have an expert human examiner monitor every participant. The question of the present work was therefore whether this same functionality could be automated using inexpensive commercial technology—specifically, whether a computer system could be designed that is capable of detecting lapses in concentration autonomously, on a trial-by-trial basis, with no input from a human operator.

## The present study

An experienced researcher or clinician will exploit a wide range of cues in order to judge when a child is concentrating on the task. These may include facial expressions, eye movements (both voluntary and reflexive), body language, and vocalizations (Mayer et al., 1995). Similarly, a wide range of physiological cues have been suggested as potential biomarkers of concentration/vigilance (see the Discussion section). However, this initial study concentrated on only a single cue: postural instability, under the assumption that an individual who is concentrating and engaged with the task will tend to sit more still than one who is not.

Postural instability (or “fidgeting”) was chosen as the focus for two main reasons. First, previous data indicated that it was relatively likely to show a measurable effect. This intuition was supported by previous data. For example, fidgeting in children has been associated previously with poorer attainment on tests of cognitive ability (Pellegrini & Davis, 1993) and greater response variability on clinical measures (Manny, Hussein, Gwiazda, & Marsh-Tootle, 2003), whereas in psychophysical experiments “restlessness” is often as cited as grounds for exclusion (Haggerty & Stamm, 1978; Mayer & Dobson, 1980). The second reason was practical. As will be discussed below, postural instability can be measured using hardware that is easy to set up, inexpensive, and robust, and these factors were considered important if the approach is to have any real-world utility. Note, however, that the claim here is not that postural instability is the only, or necessarily the best, way to detect lapses in concentration, and alternative measures are considered in the Discussion.

**Fig. 2:**
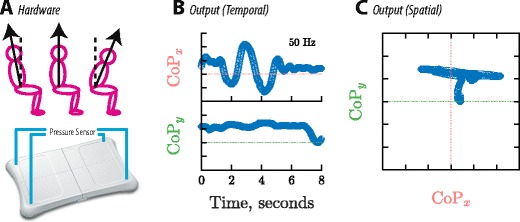
Apparatus for measuring postural instability (variability in center of pressure). (A) The hardware consisted primarily of a Nintendo Wii Balance Board: a set of four pressure sensors operating at ~50 Hz, designed to measure CoP (an approximation of a body’s center of mass, projected vertically onto the floor below). (B) Example output for a single trial, given in the time domain. (C) Same data as in panel B, but this time in the spatial domain. In the trial depicted here, the observer swayed from side to side and then leaned back

Postural instability was measured using a Wii Fit Balance Board (Nintendo Co. Ltd, Kyoto, Japan): an inexpensive gaming device that resembles a set of bathroom scales, and contains four pressure sensors designed to measure body position (see Fig. 2). This device has enjoyed considerable interest as a tool for low-cost balance assessments and rehabilitation (Goble, Cone, & Fling, 2014; Hammond, Jones, Hill, Green, & Male, 2014; Jelsma, Ferguson, Smits-Engelsman, & Geuze, 2015; Jelsma, Geuze, Mombarg, & Smits-Engelsman, 2014; Smits-Engelsman, Jelsma, Ferguson, & Geuze, 2015) and has been shown to have an accuracy comparable to that of commercial force plates (Wikstrom, 2012). It was also of particular interest because postural instability in general (Arroyo et al., 2009; Gunes, Shan, Chen, & Tian, 2015; Karg et al., 2013; Kleinsmith & Bianchi-Berthouze, 2013; Mota & Picard, 2003), and the data from the Wii Fit in particular (Clinton, D’Mello, & van den Broek, 2012), have already been shown to be productive indicators of task engagement in adults. For example, Clinton, D’Mello, and van den Broek found that undergraduate students who exhibited less postural instability while reading text tended to be better at recalling the details subsequently.

The primary output of the Wii Balance Board is a measure of the body’s center of pressure (CoP), sampled at a rate of ~50 Hz (see Fig. 2C). The prediction was that, on a trial-by-trial basis, an increase in CoP variability would be associated with a loss of concentration, where a loss of concentration was defined formally as any trial on which the observer was able to see the stimulus but responded incorrectly (a “lapse”). Lapses were measured empirically using false-negative catch trials: clearly visible (suprathreshold) stimuli, which all observers would be expected to respond to correctly. Any incorrect responses to catch trials was scored as a lapse, and the proportion of such lapses was computed as the observer’s overall lapse rate. In short, it was expected that lapses would be more frequent on trials on which postural instability (i.e., variability in CoP) was greater.

## Method

### Participants

The participants were 35 children ages 8–11 years (*M* = 9.4 years), and 34 adults aged 18–30 (*M* = 21.7 years). Children were recruited both from the UCL Child Vision Lab volunteers database, and from local primary schools, and received certificates and small prizes for participation. Adults were recruited from the UCL Psychology Subject Pool, and received monetary compensation (£7.5/h). Informed written consent was obtained from all participants (adults) or the responsible caregiver (children), and children gave their verbal assent to participate. The study was conducted in accordance with UCL Research Ethics Committee (#1960/005).

### Apparatus

Stimuli were generated in Matlab (The MathWorks Inc., Natick, Massachusetts, USA) using PsychToolbox (Brainard, 1997; Pelli, 1997) and were presented on a Sony SDM-S94 19-in. monitor (Sony Kabushiki Kaisha, Tokyo, Japan). The display measured 23.7° horizontally (1,280 pixels) and 19.0° vertically (1,024 pixels) and was located 90 cm from the observer, in a dimly lit room. Responses were entered using a keyboard, and the experimenter was seated at an adjacent table.

During testing, participant sat on a Nintendo Wii Fit Balance Board (Fig. 2A), which was placed atop an ordinary office chair and controlled remotely via Matlab, using a modified version of the WiiLab toolbox (Brindza, Szweda, Liao, Jiang, & Striegel, 2009). As part of the present work, the code used to interface with the Balance Board has been made freely available online under an open source license: https://github.com/petejonze/wiibalance. The website includes instructions on how to set up the necessary hardware and minimal working examples of its use.

To ensure that participants sat/behaved naturally, participants were not told that the Balance Board was part of the experiment, and there was no physical connection between the Balance Board and the test computer (NB: the Balance Board is interfaced with wirelessly using Bluetooth). Note that the board is intended to be stood rather than sat upon. The precise effect of sitting on the accuracy and precision of the CoP measurements is unknown, but piloting confirmed that the Balance Board remained sensitive to body movements even while participants were seated, and it was able to detect slight adjustments in posture.

## Psychophysical task and stimuli

The specific task is unimportant for the present work and is reported in more detail elsewhere (Christensen, Bex, & Fiser, 2015). In brief, observers were asked to judge which of two images contained greater orientation noise (2AFC). Each trial commenced with a zero-noise reference image, followed by two noisy images, one containing a fixed pedestal of additive orientation noise, and the other containing a variable amount of additive noise (under the control of the QUEST adaptive algorithm). The stimuli themselves consisted of “synthetic natural images”: random noise patterns that reproduced the statistics (contrast, position, phase, and orientation) of natural images. These images were constructed by summing together Gabor elements, using code described previously by Christensen, Bex, and Fiser. The task and stimuli were intended to be representative of a “typical” psychophysical experiment.

## Procedure

On each trial, observers started by viewing a random reference image, which was presented above a central fixation point for 1 s. After a 500-ms delay, the observer then viewed two noisy variants of the reference image (pedestal and target), which were presented together side-by-side below the fixation point, for 1 s. The pedestal image contained all the Gabor elements of the reference image, but the orientation of each was randomly perturbed by a sample of additive orientation noise, drawn from a normal distribution with zero mean and an *SD* of *σ*_*p*_, where *σ*_*p*_ was one of five possible values: {2, 4, 8, 26, 32}. The target image was identical to the pedestal image, except that the *SD* of the normal distribution from which the orientation of each element was drawn was increased by Δ*σ*, where the value of Δ*σ* was determined by the QUEST adaptive staircase. All stimuli were presented with raised cosine spatiotemporal envelopes. The spatial windows were circular and subtended 4° in radius with edges smoothed over 0.5°. Image contrast was ramped on and off over three video frames (50 ms).

The observer’s task was to make a 2AFC decision whether the noisier image (the “target”) was on the left or the right. Visual feedback was provided after each trial, consisting of a green light following a correct response, and a red light following an incorrect response. Further feedback was also presented at the end of each block, and was determined by performance on suprathrehhold catch trials (see below). Participants were show a “happy face” if they scored ≥80% correct, or a “sad face” otherwise. For every “happy face,” participants also won a token, and these tokens were exchanged for small prizes and certificates at the end of the experiment.

Participants completed 225 test trials, arranged into 14 blocks (“levels”). Test trials were used to determine the observer’s 75% discrimination threshold, and the stimulus magnitude (i.e., the difference in orientation noise between target and pedestal) on each trial was determined by the QUEST procedure.

In addition to test trials, observers also completed 51–59 (*M* = 55) suprathreshold (“false negative”) catch trials. These occurred every six to nine trials, with the exact trial number determined by a uniform-random distribution. Catch trials were used to measure lapse rates, and the stimulus magnitude was fixed at +30°. This magnitude was substantially greater than the just noticeable differences reported previously for adults (Christensen et al., 2015) or observed in the present study for children (*M* = 11.5, 95% CI = 8.4–24) or adults (*M* = 9.2, 95% CI = 7.1–13.3). All observers would therefore be expected to answer all catch trials correctly on every trial. Note that it is these catch trials that are of primary interest in the present study; the data from the test trials will be reported in more detail elsewhere (Dekker et al., unpublished).

Prior to testing, observers also completed 20 practice trials, containing progressively more difficult stimuli. All observers were able to complete these trials with minimal difficulties, and to the satisfaction of the experimenter.

The whole test session lasted approximately 45 min, including optional breaks between blocks, which participants were encouraged to take as required.

## Measures (molecular)

The following metrics were computed on a trial-by-trial basis, yielding one value per catch trial (i.e., *~*55 values per participant):

- ***Lapses*** are the current “gold standard” measure of inattentiveness. A lapse was defined as an incorrect response on a (suprathreshold) catch trial. The stimulus magnitude on catch trials was considerably higher than both the expected threshold and the empirical thresholds for all observers (see above). An incorrect response on a catch trial was therefore considered good evidence of a loss of concentration. Note, however, that since the task was 2AFC, it was possible for an observer to lose concentration but still to answer correctly by chance (i.e., with a probability of 50%).
- ***CoP***_***MAD***_ was the proposed new measure of inattentiveness based on postural instability. CoP_MAD_ was measured as the median absolute deviation [MAD] in CoP values on a given trial, thus:

$$ {\mathrm{CoP}}_{\mathrm{MAD}}=\mathrm{median}\left(\sqrt{{\left[{\mathrm{CoP}}_x-\mathrm{median}\left({\mathrm{CoP}}_x\right)\right]}^2+{\left[{\mathrm{CoP}}_y-\mathrm{median}\left({\mathrm{CoP}}_y\right)\right]}^2}\right) $$

MAD provides a measure of statistical dispersion, so CoP_MAD_ indicates how much the observer’s body position varied within a single trial. To make the measure of CoP_MAD_ more robust, the value on each trial was mean-averaged with the values from the preceding *N* trials. This operation is generally referred to as a simple moving average (SMA) and is equivalent to smoothing the data using a low-pass filter. For the data presented in the present article, *N* was fixed at 2, but other values of *N* yielded qualitatively similar results/conclusions to those reported here (see the supplemental materials).

## Measures (molar)

The following metrics were computed across an entire test session, yielding one value per participant:

- ***Threshold*** provided a summary measure of sensitivity. Threshold was computed as the smallest stimulus magnitude that the participant was able to detect with 75% reliability, as estimated by the mean of the QUEST posterior density function.
- ***Lapse rate*** provided a summary measure of inattentiveness. Lapse rate was computed as 2*L*, where *L* is the proportion of catch trials on which lapses occurred and 2 is a correction for the 2AFC nature of the task. An ideal observer would be expected to produce a lapse rate of 0. A maximally inattentive observer would be expected to produce a lapse rate of 1.
- ***Mean CoP***_***MAD***_ provided a summary measure of postural instability. Mean CoP_MAD_ was computed as the arithmetic mean of all CoP_*MAD*_ values for a single observer.
- ***Experimenter attentiveness rating*** provided a secondary summary measure of inattentiveness. These ratings were made by the experimenter at the end of each session and were scored on a scale of 1 (*very inattentive*) to 5 (*very attentive*). Due to human error, scores were recorded for only 57 of 74 participants.

## Analysis

The data were not normally distributed, so they were analyzed and reported using nonparametric techniques (e.g., Wilcoxon signed-rank test, Spearman correlation). All error bars represent 95% confidence intervals (CI_95_) and were derived using bootstrapping (DiCiccio & Efron, 1996)—bias-corrected and accelerated percentile method (*N* = 20,000).

## Results

### Analysis of trial-by-trial postural instability data

**Fig. 3:**
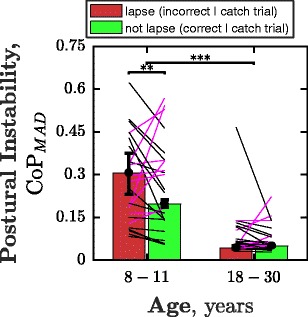
Median (± CI_95_]) postural instability across trials (CoP_MAD_), measured as a function of both age and whether or not a lapse was observed. Lines show the data for individual observers and are color-coded on the basis of whether CoP_MAD_ was greater (black) or smaller (magenta) on lapse trials

Figure 3 shows how postural instability, as measured by CoP_MAD_, varied between catch trials on which lapses did and did not occur. For children, CoP_MAD_ was significantly greater on incorrect catch trials (Wilcoxon signed-rank; *Z* = 2.86, *p* = *.*004), meaning that children shifted their posture to a greater extent on trials where lapses in concentration occurred. This qualitative pattern was observed in 68% of children (Fig. 3, black lines), although a substantial minority of individuals showed the inverse pattern (Fig. 3, magenta lines). There was also a significant main effect of age (Z = 40.34, *p* ≪ .001), with children exhibiting greater postural instability than adults.

**Fig. 4:**
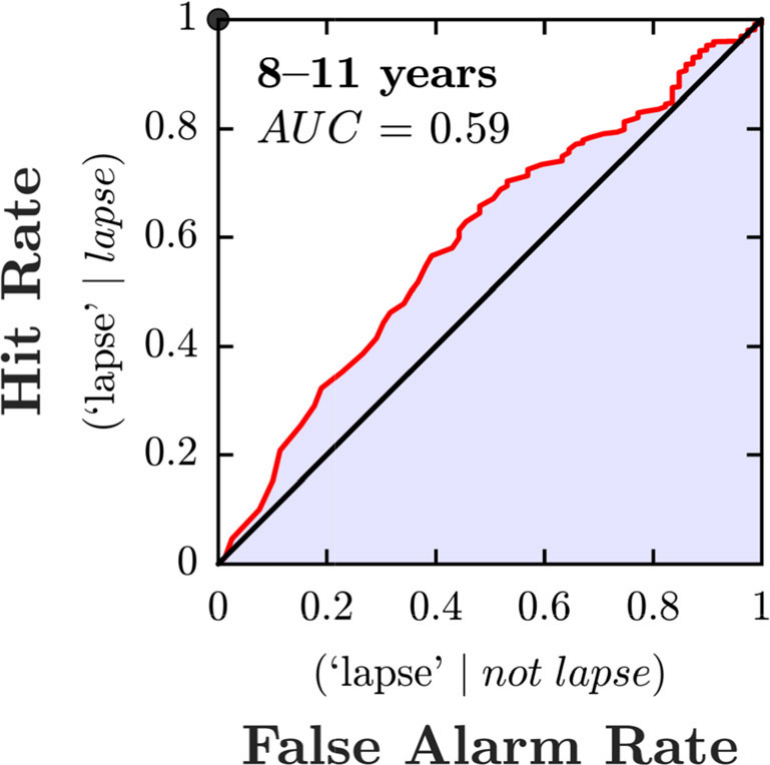
Receiver operating characteristic (ROC) using CoP_MAD_ to classify catch trials as “lapse” or “no lapse.” The ideal classifier is shown at top left (0, 1), and the diagonal line denotes chance. The area under the curve (AUC) is .59

To further assess the balance board’s effectiveness at identifying lapse trials, children’s empirical data were used to construct a receiver operating characteristic (ROC; Fig. 4). An ideal classifier would yield a point at the upper left corner, or coordinate 〈0, 1〉 of the ROC space, meaning 100% sensitivity (no false negatives) and 100% specificity (no false positives). In contrast, it is clear by inspection that CoP_MAD_ is an imperfect classifier: Any criterion would lead to genuine lapses in concentration being missed and correct responses being misclassified as lapses. However, the ROC is also clearly distinguishable from the diagonal line of no discrimination, indicating that CoP_MAD_ does contain useful information. Note also that this analysis provides only a lower bound on the method’s effectiveness, since the reference tags themselves were subject to measurement error (i.e., due to the 2AFC nature of the task, observers will have answered correctly by chance on some trials even when lapses in concentration did occur, meaning that some true lapses will have been tagged incorrectly as *not lapses*).

### Between-subjects analyses

**Fig. 5:**
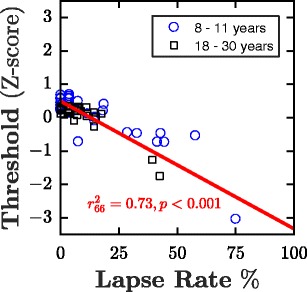
Estimated thresholds as a function of lapse rate. Each marker indicates an observer. The red line shows the best-fitting linear regression slope (one outlier excluded: 〈8.8, −6.9〉). Threhsolds are given as *Z* scores, in the same manner as in Fig. 1

The median lapse rates were 1.9% for both children and adults, with no significant difference between the two age groups (Wilcoxon signed-rank: Z = – 0.35, *p* = *.*729). Similarly, there was no significant difference in median thresholds between children and adults (Z = 1.67, *p* = *.*094). Across all participants, however, a significant linear relationship emerged between lapse rate and threshold [*r*^2^(66) = .73, *p* < .001], as shown in Fig. 5. This pattern of results also held true when regressions were performed separately in children [*r*^2^(33) = .82, *p* < .001] and adults [*r*^2^(32) = .75, *p* < .001]

Average postural instability, as measured by mean CoP_MAD_, decreased (improved) with age (Z = 5.64, *p* < .001). In children, there was no significant relationship between mean CoP_MAD_ and mean lapse rate [Spearman correlation, *r*(33) = .06, *p* = *.*148], estimated threshold [*r*(33) = .07, *p* = *.*124], or experimenter ratings of inattentiveness [*r*(17) = .12, *p* = *.*149]. In adults, weak, borderline relationships were observed between mean CoP_MAD_ and both mean lapse rate [*r*(32) = .11, *p* = *.*058] and estimated threshold [*r*(32) = .12, *p* = *.*042], but there continued to be no significant relationship between mean CoP_MAD_ and experimenter ratings of inattentiveness [*r*(31) < .01, *p* = *.*744]. Taken together, these results indicate that average postural instability is not an effective measure of an individual’s overall level of attentiveness, particularly in children. Note that the absence of any strong relationships did not appear to be due to measurement error (i.e., intrinsic variability in the estimates of mean CoP_MAD_). Thus, a split-half analysis (odd vs. even trial numbers) indicated that estimates of mean CoP_MAD_ were relatively consistent within both individual children [*r*(33) = .83, *p* < .001] and adults [*r*(32) = .65, *p* < .001].

## Discussion

This study examined whether postural instability, as measured by the Wii Fit Balance Board, can be used to detect lapses in concentration during psychophysical testing. The results were encouraging, in that children exhibited greater postural instability (higher CoP_MAD_) on catch trials on which lapses occurred. This indicates that postural instability does provide a real-time index of concentration. That fidgeting is a correlate of poorer performance is consistent with our everyday intuitions, as well as with a number of previous studies that have reported links between fidgeting in children and poorer performance on tests of cognitive and perceptual abilities (Manny et al., 2003; Pellegrini & Davis, 1993).

However, the data also raised important concerns. A formal ROC analysis of the trial-by-trial data showed that, as a predictor of lapses, postural instability had limited sensitivity and specificity, and further refinement of the measure is required (see below). The trial-by-trial relationship between CoP_MAD_ and lapses was not observed in adults, who sat relatively still throughout testing. Nor was postural instability an accurate predictor of a child’s overall attentiveness, with mean CoP_MAD_ failing to correlate either with overall lapse rates or with experimenter ratings. Below, we will consider how the information provided by an automated “lapse detector” might be used in practice, how the measure might made more robust, and how the present approach could be extended in future.

### Benefits, implications, and potential applications

The current gold standard metric of inattentiveness is lapse rate: the proportion of incorrect responses on false-negative catch trials. Computing lapse rates requires additional, dedicated catch trials, which prolong overall test duration, and so can be potentially counterproductive in terms of ensuring the child remains engaged throughout testing. Estimates of lapse rate also tend to be relatively inaccurate, since lapses are rare events and so require a large number of catch trials to estimate accurately (see Wichmann & Hill, 2001). Finally, lapse rates are a “molar” measure (Green, 1964), and provide no indication on which specific trials the child was concentrating. At best, they can therefore only be used to exclude participants post hoc. In contrast, the proposed new approach does not require additional trials (i.e., runs concurrently during test trials) and allows lapses to be identified and responded to on a trial-by-trial (“molecular”) basis.

The most straightforward use for such a measure would be to exclude or repeat “bad” trials, as is currently done in some existing clinical tests when a human operator notices a lapse in concentration (see Introduction). Alternatively, an observed loss of concentration could be used to trigger an active countermeasure, such as short breaks, explicit encouragement, or easy “motivational” trials. Finally, a third—and potentially more efficient—use for such data, would be to combine estimates of inattentiveness probabilistically with modern psychophysical algorithms, such as QUEST, QUEST+, or Psi. To see how this could be achieved, note that such algorithms fundamentally use Bayes’s theorem to compute a posterior probability density for each parameter of an arbitrary psychometric model, *ψ*, given a vector of trial-by-trial stimulus values, **x**, a vector of trial-by-trial observer responses, **r**, and a set of priors, *P*(*ψ*). Thus:

$$ P\left(\left.\psi \right|\kern0.5em \left\{\mathbf{x},\mathbf{r}\right\}\right)\propto P\left(\mathbf{r}\left|\kern0.5em \left\{\mathbf{x},\psi \right\}\kern0.5em \right.\right)P\left(\psi \right), $$

where *P*(*r* | {x,*ψ*}) represents the *likelihood* of the observed data and is given by

$$ P\left(\left.\mathbf{r}\right|\kern0.5em \left\{\mathbf{x},\psi \right\}\right)=\prod \limits_{i=1}^nP\left({\mathbf{r}}_i\left|\kern0.5em \left\{{\mathbf{x}}_i,\psi \right\}\right.\right). $$

To integrate estimates of lapses into this model, the likelihood function simply needs to be modified such that each of the observer’s responses is weighted by the probability of a lapse having occurred on that trial, *α*(*θ*_*i*_), thus:

$$ {P}^{\alpha}\left(\mathbf{r}\left|\kern0.5em \left\{\mathbf{x},\psi \right\}\right.\right)=\prod \limits_{i=1}^n\left[P{\left({\mathbf{r}}_i\left|\kern0.5em \left\{{\mathbf{x}}_i,\psi \right\}\right.\right)}^{\alpha \left({\boldsymbol{\theta}}_i\right)}\right]\kern1em where\kern1em 0\le \kern0.5em \alpha \left({\boldsymbol{\theta}}_i\right)\le 1. $$

When *α*(*θ*_*i*_) = 0 (definite lapse), that trial is given zero weight—the observer’s response is effectively ignored, and the posterior estimate remains unchanged. When *α*(*θ*_*i*_) = 1 (definite concentration), the trial information is integrated into the posterior exactly as per usual—that is, the outcome of the trial will be used to update the current state posterior density functions. At intermediate values of *α*(*θ*_*i*_), trials are given “partial credit.” This “weighting” approach has been suggested in other domains as a way of adjusting for anomalous statistical data (Agostinelli & Greco, 2012) and has been shown to provide a consistent and efficient likelihood estimate, while preserving the same first order asymptotic properties of a genuine likelihood function (see Agostinelli & Greco, 2012).

Note that under this proposed scheme, all other aspects of the psychophysical algorithm remain unchanged. It is therefore still possible, for example, to compute expected entropy, which can be used both to determine the most informative stimulus to present on the next trial, and to ascertain when a given level of measurement certainty has been attained. In this way, estimates of attentiveness could be incorporated automatically into the stopping criterion of the psychophysical algorithm, meaning that a more attentive child would be required to complete fewer trials.

The prediction is that such a probabilistic-weighting approach would yield tests that are faster, more reliable, and exhibit higher completion rates than current methods that blindly assume that all of an observer’s responses are equally informative. In particular, overall measurement variability should become smaller and more normally distributed (i.e., fewer outliers). Note that under this new scheme, a conspicuously inattentive child might never reach the stopping criterion within a prescribed number of trials, and so would be scored as “did not complete.” However, this seems preferable to the present situation, in which such individuals produce spurious data that must be excluded post-hoc, often using dubious or ineffective statistical criteria (Jones, 2016).

### Improving the detection of lapses using other measures

As a sensor, the Wii Fit Balance Board is particularly attractive due to its low cost (~£60) and ease of use (e.g., highly portable, with minimal setup time). However, the results of the present study show that Postural Instability alone is an imperfect lapse-detector. This is unsurprising, given that a human experimenter will actually use a wide range of different cues to determine when a participant is alert and engaged (Mayer et al., 1995). A key question for the future, therefore, is whether additional sources of information could be similarly combined within a fully automated system. For example, in adults it has been suggested variously that eye movements (D’Mello, Olney, Williams, & Hays, 2012), head movements (Westlund, D’Mello, & Olney, 2015), movements of the upper body and torso (Sanghvi et al., 2011), skin conductance/temperature (Blanchard, Bixler, Joyce, & D’Mello, 2014), heat rate (Libby, Lacey, & Lacey, 1973), vocal expressions (Meng & Bianchi-Berthouze, 2014; Metallinou, Katsamanis, & Narayanan, 2013), facial expressions (Bosch, D’Mello, Ocumpaugh, Baker, & Shute, 2016; Whitehill, Serpell, Lin, Foster, & Movellan, 2014), self-reports (McVay & Kane, 2012b; Schooler, Reichle, & Halpern, 2004), response time latency (McVay & Kane, 2012a; Unsworth & Robison, 2016), response time variability (Esterman, Noonan, Rosenberg, & DeGutis, 2013; Esterman, Rosenberg, & Noonan, 2014), double-pass response consistency (Burgess & Colborne, 1988; Green, 1964), EEG-based neural activity (Adam, Mance, Fukuda, & Vogel, 2015; Davidson, Jones, & Peiris, 2007; Jung, Makeig, Stensmo, & Sejnowski, 1997), fMRI BOLD responses (deBettencourt, Cohen, Lee, Norman, & Turk-Browne, 2015; Esterman et al., 2013; Esterman et al., 2014; Rosenberg et al., 2016), and/or pupil dilation (Libby et al., 1973; Unsworth & Robison, 2016; van den Brink, Murphy, & Nieuwenhuis, 2016) could each be used as an indicator of whether the participant is alert and engaged (for overviews, see D’Mello, Dieterle, & Duckworth, 2017; Gunes & Schuller, 2013; Kleinsmith & Bianchi-Berthouze, 2013).

Of these, some sensors are more practicable than others. Thus, measures of response latency or consistency can be readily computed without any specialized equipment, whereas fMRI-based metrics are impractical for most everyday scenarios, and are likely to remain so for the foreseeable future. Other proposed measures fall between these two extremes in terms of cost and complexity. For example, remote eye-tracking could be used to ensure that participants are fixating on the stimulus area, video-refraction could be used to ensure the eyes are focused and converged, head-pose tracking could be used to detect subtle movements in the head or shoulders, and face recognition could be used to register facial expressions and emotions. Encouragingly, although these measures do sometimes require additional hardware, the requisite technology is becoming increasingly cheap and accessible. For example, the Kinect 360 sensor for head and face tracking (Microsoft, Redmond, Washington, USA) or the Tobii EyeX eye-tracker (Tobii AB, Danderyd, Sweden) can both be purchased commercially for less than £100. Like the Wii Fit Balance Board, these devices are intended primarily for home gaming purposes but can be easily modified for psychophysical testing. Even more excitingly, many of these measures, such as head-pose and face tracking, can now even be performed reliably using ordinary optical sensors, combined with advanced image-processing algorithms (Baltrušaitis, Robinson, & Morency, 2016). It would be extremely interesting to compare directly the efficacy of these various approaches in cohorts of children and adults.

Assuming additional measures can be identified, the next challenge will be to integrate the various sources of information together, to provide a single, overall index of task engagement. Here again, though, recent technological developments augur well. Thus, using modern machine learning techniques (e.g., multilayer perceptrons, support vector machines, or related linear classifiers; see Bishop, 2007), it should be relatively straightforward to combine information of different formats together, and to weight each channel appropriately (i.e., in proportion to the reliability of its source). The hope is that, together, these measures will provide a more accurate means of detecting lapses than any single measure could alone: a fact that could be evidenced by, for example, a steepening of the ROC in Fig. 4.

### Key limitations and future work

Participants in the present study performed a 2AFC task. A 2AFC design was used because the goal was to model a typical psychophysical experiment, and this classic “Fechnerian” method remains the most prevalent one in the literature. However, it also meant that many genuine lapses in concentration likely went undetected, and, as a consequence, the predictive relationship between Postural Instability and the occurrence of lapses may have been underestimated (see the Results section). A more accurate estimate of lapse rates could be achieved in future by simply increasing the number of response alternatives, in order to minimize the likelihood of false positive (“correct guess”) responses. Doing so should not qualitatively change any of the present conclusions, but may make the observed effect sizes greater (i.e., increase the area under the curve in Fig. 4).

The present study was concerned solely with the detection of lapses: dichotomous events that either succeed or fail to occur. Intuitively, however, lapses are only one extreme end of a continuum. One can concentrate on, or “attend to” a task to a greater or lesser degree^1^ (White, 1964), and an observer can be said to be more or less careful or vigilant. Consistent with this, fMRI correlates of performance on tasks requiring sustained attention have been reported to fluctuate gradually between different states of activity (Esterman et al., 2013; Esterman et al., 2014; Rosenberg et al., 2016). In this sense, lapses can be thought of as only the final manifestation of some more gradual process, and further benefits might be gained by moving beyond simple lapse detection and instead attempting to track the putative fluctuations in concentration that underlie them. For instance, it may be that smaller changes in concentration exist that are not sufficient to produce errors on suprathreshold stimuli (lapses), but that are nonetheless capable of degrading perceptual judgments (e.g., one might imagine some mechanism of attention that flattened the slope of the psychometric function, rather than lowering its upper asymptote). In this case, the ability of to track more gradual changes in concentration could be instrumental for partialing out cognitive factors to obtain a “pure” measure of sensory ability. Alternatively, it may be that preventative interventions, such as breaks, feedback, or encouragement, are more effective when delivered early, when concentration is only just starting to wane. These are highly complex questions, however, and answering them will ultimately require us to devise more nuanced “ground-truth” measures, capable of indexing incremental fluctuations in attentiveness. This might be attempted, for example, by moving away from traditional psychophysical tasks with binary outcomes (correct, incorrect), toward tasks with continuously distributed outcomes, such as score or response latency. Alternatively, it might be instructive to look not only at whether better correlates of lapses can be devised (see above), but also at whether more gradual, cumulative changes can be discerned on those trials preceding lapses.

### Conclusions

A strong negative relationship was observed between lapse rates and estimated thresholds, both in the present data and when reanalyzing data from previous studies. This confirms that lapses in concentration are a substantive confounding factor when attempting to measure perceptual thresholds. Such confounds have the potential to explain many individual differences or age effects.

Among 8- to 11-year-old children, there was a significant difference in postural instability (CoP_MAD_) between trials on which lapses did or did not occur, with children exhibiting greater movement on trials on which a loss of concentration (lapses) occurred. This indicates that postural instability (“fidgeting”) can be used to discriminate between trials on which the child was or was not concentrating. Such measurements could in future prove instrumental in improving the quality of psychophysical measurements and/or for better understanding individual differences in performance.

A formal ROC analysis confirmed that postural instability (CoP_MAD_) is a better-than-chance predictor of lapses. However, sensitivity and specificity were limited when using postural instability alone (AUC = .59). Other potential sources of additional information were discussed, which might be provide better measures, or which could be combined with postural instability to improve detection rates.

A simple method was presented for incorporating estimates of attentiveness into modern (Bayesian/maximum-likelihood) psychophysical procedures, by weighting each response by the probability of a lapse having occurred on that trial.

Overall, the results demonstrate that the proposed approach is feasible, but that postural instability alone is an imperfect index of lapses. The goal for the future is to refine the approach to produce an autonomous system that is as accurate as an expert human experimenter at judging when a child is alert, engaged, and compliant.

## Electronic supplementary material

ESM 1 (PDF 147 kb)

## Footnotes

1 Although, as is discussed by White (1964), to concentrate more or less does not necessarily imply “more or less intense engagement in one specific activity, but rather as concentrating more or fewer of our activities on the one object” (p. 7).
