# Supplementary material for: Sit still and pay attention: Using the Wii Balance-Board to detect lapses in concentration in children during psychophysical testing
Source: Behav Res Methods. 2018 May 16;51(1):28–39. doi: 10.3758/s13428-018-1045-4 (PMC6420608; doi:10.3758/s13428-018-1045-4)
Supplement: Supplementary file 1 — (PDF 147 kb) [file 13428_2018_1045_MOESM1_ESM.pdf]

Supplemental Material: Sit still and pay attention: Using the Wii Balance-Board to detect lapses in concentration in children during psychophysical testing

Pete R. Jones

1. Smoothing parameter when computing Postural Instability (CoP_{MAD})

Trial-by-trial estimates of Postural Instability (CoP_{MAD}) were smoothed using a Simple Moving Average (SMA), in which the value on each trial was mean-averaged with the values from the preceding N trials. For the data presented in the main manuscript, N was fixed at 2. However, as shown in **Fig S1**, other values yielded qualitatively similar results/conclusions.

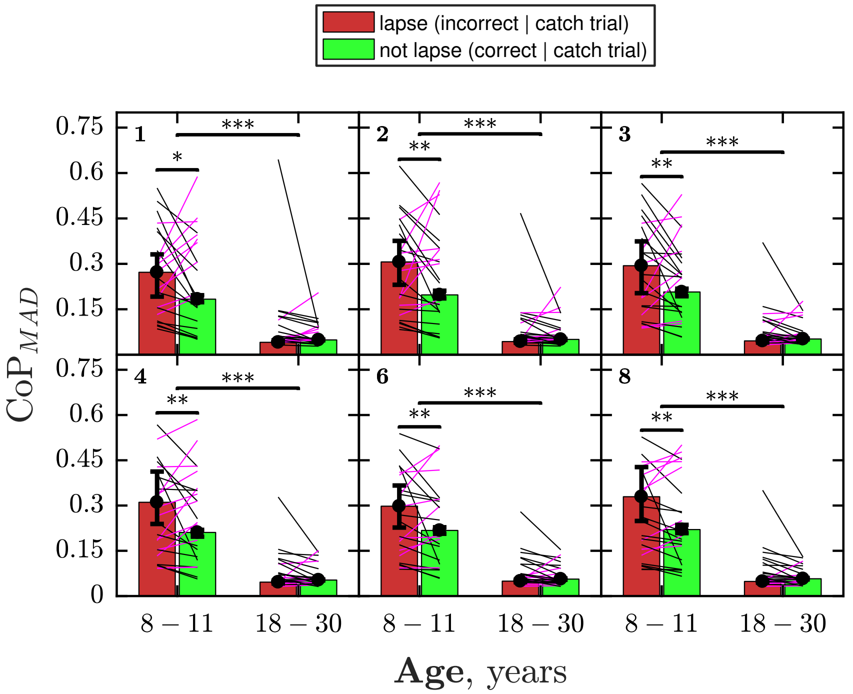

Fig S1. Median [$\pm CI_{95\%}$] Postural Instability across trials (CoP_{MAD}), presented in the same format as Figure 3 in the main manuscript. Each panel represents a different smoothing parameter (N), as shown in the top-left of each panel.
